# Exercise intervention for unilateral amputees with low back pain: study protocol for a randomised, controlled trial

**DOI:** 10.1186/s13063-017-2362-0

**Published:** 2017-12-29

**Authors:** Joseph G. Wasser, Daniel C. Herman, MaryBeth Horodyski, Jason L. Zaremski, Brady Tripp, Phillip Page, Kevin R. Vincent, Heather K. Vincent

**Affiliations:** 1 Department of Orthopaedics and Rehabilitation, UF Health Orthopaedics and Sports Medicine Institute (OSMI), University of Florida, Gainesville, FL 32611 USA; 2 Department of Applied Physiology and Kinesiology, University of Florida, Gainesville, FL 32608 USA; 3 Performance Health, Baton Rouge, Louisiana, USA

**Keywords:** Amputee, Gait, Low back pain, Physical function, Disability, Randomised controlled trial

## Abstract

**Background:**

Atraumatic lower limb amputation is a life-changing event for approximately 185,000 persons in the United States each year. A unilateral amputation is associated with rapid changes to the musculoskeletal system including leg and back muscle atrophy, strength loss, gait asymmetries, differential mechanical joint loading and leg length discrepancies. Even with high-quality medical care and prostheses, amputees still develop secondary musculoskeletal conditions such as chronic low back pain (LBP). Resistance training interventions that focus on core stabilization, lumbar strength and dynamic stability during loading have strong potential to reduce LBP and address amputation-related changes to the musculoskeletal system. Home-based resistance exercise programs may be attractive to patients to minimize travel and financial burdens.

**Methods/design:**

This study will be a single-assessor-blinded, pre-post-test randomised controlled trial involving 40 men and women aged 18–60 years with traumatic, unilateral transtibial amputation. Participants will be randomised to a home-based, resistance exercise group (HBRX) or a wait-list control group (CON). The HBRX will consist of 12 weeks of elastic resistance band and bodyweight training to improve core and lumbopelvic strength. Participants will be monitored via Skype or Facetime on a weekly basis. The primary outcome will be pain severity (11-point Numerical Pain Rating Scale; NRS_pain_). Secondary outcomes will include pain impact on quality of life (Medical Outcomes Short Form 36, Oswestry Disability Index and Roland Morris Disability Questionnaire), kinematics and kinetics of walking gait on an instrumented treadmill, muscle morphology (muscle thickness of multifidus, transversus abdominis, internal oblique), maximal muscle strength of key lumbar and core muscles, and daily step count.

**Discussion:**

The study findings will determine whether a HBRX program can decrease pain severity and positively impact several physiological and mechanical factors that contribute to back pain in unilateral transtibial amputees with chronic LBP. We will determine the relative contribution of the exercise-induced changes in these factors on pain responsiveness in this population.

**Trial registration:**

ClinicalTrials.gov, ID: NCT03300375. Registered on 2 October 2017.

**Electronic supplementary material:**

The online version of this article (doi:10.1186/s13063-017-2362-0) contains supplementary material, which is available to authorized users.

## Background

Amputation to the lower extremity is a life-changing event. Approximately two million Americans live with limb loss. An estimated 45% of these amputations are caused by traumatic mechanisms [1, 2]. Once the injury has healed and a prosthetic limb is fitted, the long-term care focuses on maintenance of the prosthesis fit and optimizing physical function [3]. However, even with the use of high-quality prostheses, biomechanical symmetry of gait remains permanently altered [4]. A unilateral transtibial amputation leads to progressive skeletal muscle atrophy in the affected lower extremity [5] and back [6]. The collective effects of asymmetric gait, loss of muscle mass, and reduction of strength increase mechanical stresses at the lumbar spine [4, 7]. These stresses contribute to low back pain (LBP) onset. LBP is a secondary complication in over half of the unilateral amputee population [8–10]. LBP interferes with physical and mental well-being and overall quality of life (QOL) [10].

Long-term care for amputees requires a team of physicians, prostheticians, therapists, and other specialists. Patients often travel long distances to receive their care, and many have resource limitations. Patients may not be able to maintain consistent long-term relationships with therapists or purchase fitness memberships due to financial constraints. Effective home-based interventions that target LBP could minimize travel or cost burden to the patient and substantially impact the secondary disease burden. An exercise mode with strong potential for a home-based intervention is resistance exercise. In the general population, resistance exercise is associated with lower odds of developing LBP [11]. Resistance exercise increases lumbar muscle strength, physical functional, and both physical and mental aspects of QOL [12–17]. The impact and effectiveness of resistance training on chronic LBP severity and pain impact in amputees is not yet known. Moreover, the mechanisms that may contribute to the effectiveness of strength training on LBP relief in amputees are not clear. These evidence gaps are significant barriers to the optimization of care for this special population.

The aims of this study are to determine whether home-based resistance exercise (HBRX) among unilateral transtibial amputees with LBP can reduce LBP severity and its impact on QOL and improve the biomechanical symmetry of gait. The relative contribution of key factors that predict pain responsiveness with HBRX will be determined. The research hypotheses are as follows: (1) HBRX will reduce subjective pain ratings, pain medication use, and the impact of pain on QOL; (2) HBRX will reduce gait asymmetries and increase gait velocity; and (3) HBRX-induced changes in lumbar muscle strength, lumbar muscle cross-sectional area, and perceived QOL will predict pain responsiveness for LBP in unilateral transtibial amputees.

## Methods

### Trial design

**Fig. 1:**
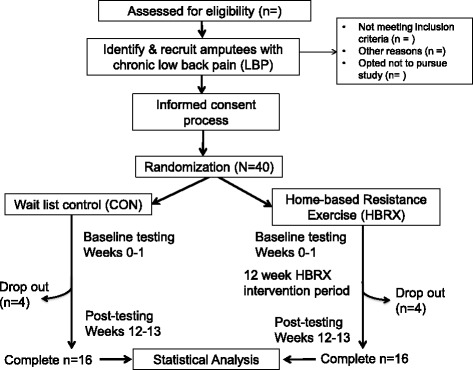
Study flow diagram

**Fig. 2:**
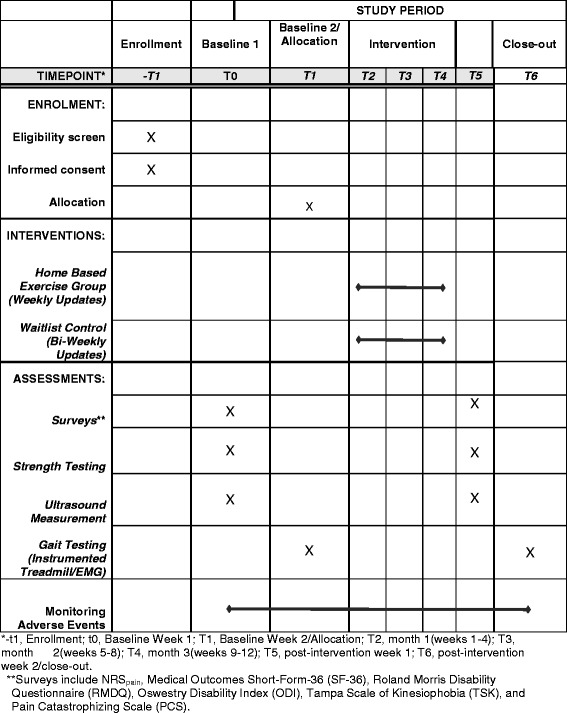
Schedule of enrollment, interventions, and assessments

This study will be a single-assessor-blinded, pre-post-test randomised controlled trial. The study will follow the principles of the Consolidated Standards of Reporting Trials for randomized, two-group, parallel studies [18, 19]. Figure 1 provides the study flow and Fig. 2 shows a version of the Standard Protocol Items: Recommendations for Interventional Trials (SPIRIT) Figure for the trial. Details of study methodology are outlined in Additional file 1, SPIRIT Checklist. There will be an HBRX intervention group and a wait-list control group (CON). Outcome measurements will be performed before the intervention period (baseline assessment) and immediately after the 12-week intervention (post-testing assessment). Baseline and follow-up measurements will be evaluated blindly.

### Ethical aspects

This study and its procedures were approved by the University of Florida Institutional Review Board (UF IRB) (protocol number 201701256). Although unanticipated, any changes to the protocol and study procedures will be requested through UF IRB and subsequently to ClinicalTrials.gov and the study sponsor. Written informed consent will be obtained from all candidates who agree to participate and meet all the inclusion/exclusion criteria. Data will be used only in aggregate and no identifying characteristics of individuals will be published or presented. Confidentiality of data will be maintained by using research identification (ID) numbers that uniquely identify each individual. Safeguards will be established to ensure the security and privacy of participants’ study records. Appropriate measures will be taken to prevent unauthorized use of study information. Data other than demographic information do not use names as an identifier. The research ID number will be used. The research records will be kept in a locked room on site. The files matching participants’ names and demographic information with research ID numbers will be kept in a separate room and will be stored in a locked file that uses a different key from that of all other files. Only the study team members will have access to these files, and they will be asked to sign a document that they agree to maintain the confidentiality of the information. Only the study team members and the UF IRB will have access to the full data set.

### Study population

A total of 40 transtibial amputees aged between 18 and 65 years will be recruited for this study. Participants will be recruited from a major regional prosthetics center and from the greater north Florida community via relationships with local prosthetic clinics and amputee support groups. Study flyers, social media posts, and online web advertisements will also be used. Participants will be eligible if they meet the following inclusion and exclusion criteria. Inclusion criteria: men or women aged 18–60 years, traumatic transtibial amputation more than 1 year prior to enrollment, current prosthesis worn for at least 6 months, suffering from chronic LBP (>3 months with at least three pain episodes per week), must have regular access to a computer for Skype®, or a mobile phone or iPAD to perform Facetime; prosthesis K-level of K2 or greater (indicating the subject is able to ambulate and traverse low-level environmental barriers such as curbs, stairs, or uneven surfaces) [20]. Exclusion criteria: dysvascular amputation, acute back injury, pregnancy, any other chronic back pathology (i.e., herniated disc, ankylosing spondylosis, other related neurological disease), pain symptoms or functional limitations (including those that may require assistive devices) that preclude participation in resistance exercise or physical activity, back surgery within the past 2 years that restricts daily physical activities, and currently enrolled in any other resistance or strengthening exercise interventions. Written informed consent will be obtained from each participant.

The primary outcome of the study is the Numerical Pain Rating Scale (NRS_pain_) value for LBP. Sample size estimation indicated that a total sample size of 32 was necessary to detect clinically meaningful differences in NRS_pain_ from baseline to week 12. Based on published evidence of comparative studies of resistance exercise and controls on LBP [21], a sample size of 16 per group will yield a power of 80% at an *α* value of 0.05. To account for the anticipated dropout rate of 25%, the enrollment target will be 40.

### Randomisation and concealment

Following baseline measures, participants will be randomised to either the HBRX or the CON group. The randomisation will be performed by a clinical coordinator not involved in the study. Permuted block randomization [22] with block sizes of 4 will be made by means of a computer algorithm to ensure balanced group sizes and allocation concealment. Patients will receive an opaque, sealed envelope taken from a sequential order containing information on group allocation.

### Home-based resistance exercise (HBRX) intervention

**Fig. 3:**
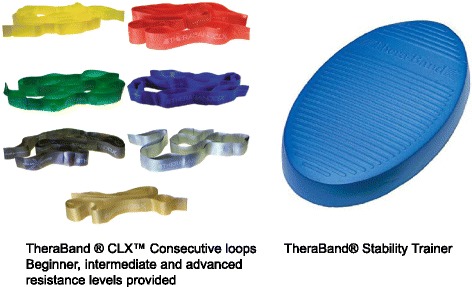
Elastic resistance band set and stability pad equipment for the home-based resistance

Participants in the HBRX group will be coached, by a qualified study team member, through six phases of the intervention with 2 weeks per phase. Table 1 summarizes the HBRX intervention. Body weight and elastic exercise bands will be used to provide resistance. The lightest resistance band is 3.7 lbs which is low enough for all qualified participants to perform with. Moreover, modifications to exercises are available to accommodate such things as pain. For all exercises, the amputees will wear their prosthetic. A set of commercial elastic resistive bands (TheraBand® CLX) and a stability trainer (Fig. 3; Performance Health, Akron, OH, USA) will be provided to each participant. The use of elastic bands for resistance training can induce results in neuromuscular adaptations and strength similar to those achieved by weight machines and free-weights [23–26].

**Table 1:** Home-based resistance exercise intervention

Exercise	Description	Progression of exercise
Plank	Prone lying static position with participant’s weight resting on their forearms while holding their body in a straight line from head to toe. Hold this position, with good form, for as long as possible	Progress to an unstable surface/instability disk
Seated resisted back extension^a^	While seated, place feet into band loops. Have participants pull end loops of the band and create an “X” in front of them. Fold and raise arms to shoulder height. Participants will bend their trunk forward at the waist and return to a “straight back” seated position	Progress to an increased resistance band level
Trunk rotary stabilization^a^	With resistance band anchored at chest level, create tension with band. While standing in line with the band, fully extend arm out to participant’s side at about 30°. Use other hand to push the band forward while maintaining stability in participant’s core. Hold this position for 2–3 s and return to starting position to repeat	Progress to an increased resistance band level
Leg extensions^a^	While standing, place feet into band loops with one seal between them. With feet hip width apart, participants will center their balance onto one leg. Keeping one leg straight, slowly raise and kick backwards without touching the ground. Keep back straight and avoid leaning or bending over. Once finished, place foot back into starting position	Progress to an increased resistance band level
Monster walks^a^	Place legs through band loops making sure they reach right above the knee. Grab the end of the resistance band and while maintaining a slight bend in the knees and hips, take 3 steps laterally while keeping back straight	Progress to an increased resistance band level
Posture reset^a^	Place each hand into CLX™ loop so that hands are one loop apart. Supinate open palms and have participant’s elbows at 90° with hands in front. Extend elbows and shoulder outward and retract shoulder blades	Progress to an increased resistance band level
Abductor resistance^a^	Place feet in the middle two loops of the resistance band. Have the participant grab handles of band and create an “X” behind their knees before pulling the band around the outside of their hips and cross their hands in front of their waist. With a slight bend in the participants’ knees, and maintaining balance, kick one leg out to the side and returning to the starting position. Repeat for opposite leg	Progress to an increased resistance band level
Supermans	While prone lying with arms and legs outstretch, slightly raise both arms and legs off the floor/table in unison	Progress to alternating contralateral arms and legs

^a^Adapted from TheraBand® CLX™ consecutive loops (http://www.therabandclx.com/exercises.html)

Based on the principles and guidelines of resistance exercise provided by the American College of Sports Medicine (ACSM), the following prescription was crafted [27–30]. Eight exercises will initially be performed 2 days per week for the first phase to familiarize subjects to the exercises and provide instruction on proper form. Exercises were chosen to emphasize core strength [31], dynamic hip and pelvis stability, and lumbar endurance and strength [32]. These exercises include plank, seated resisted back extension, trunk rotary stabilization, leg extensions, monster walks, posture reset, abductor resistance, and prone arm and leg extensions (Supermans) [6]. These exercises were based on our earlier resistance-training programs that improved low back and core strength by training these muscles [33]. For the remaining phases, exercises will be completed three times per week [20]. Participants will be performing three sets of 8–12 repetitions for all exercises, per the ACSM general guidelines for muscular hypertrophy [27–30].

The first exercise session will be performed face-to-face with the participant to allow the investigators to teach the form and make form adjustments. Exercises will be subsequently supervised via Skype® or Facetime with each participant on a weekly basis. Exercise sessions will be documented by the participant in a standard training log. Participant rating of perceived exertion values will be used to assign the band resistance level or number of repetitions for each exercise. The exercises should be perceived as an effort level of between 13 and 14 out of 20 points on the 6–20-point Borg Scale [34], or 6–7 on the Resistance Intensity Scale for Exercise (RISE) scale. The Borg Scale is used for general exercise effort, and the RISE scale was crafted specifically for use with TheraBand® elastic bands [35]. As the participant gets stronger over time, participants will be asked to perform effort levels that fall between 16 and 17 on the Borg Scale and 8 and 9 on the RISE scale. To achieve this, the study team will increase the resistance load/volume (by 2–5%) to keep the effort level at the target during the study [33].

### Wait-list control (CON) condition

Participants who are assigned to the CON group will wait to participate in the resistance training after a 12-week wait period. Participants will follow all instructions provided to them by their physician and care team, but will be asked to refrain from starting any new strengthening exercise protocols or begin any new physical therapies during this time. The participants will be contacted by phone on a monthly basis during the study period to determine if any changes in LBP symptoms, exercise schedule, or medication have occurred.

### Compliance and attrition

Treatment compliance will be assessed by recording the number of completed HBRX training sessions. Participants will be asked to keep an exercise log indicating the exercises performed and RISE and RPE scores for each exercise. Compliance will be defined as completing 85% of all sessions [20]. Members of the study team will be monitoring the exercise performance on a weekly basis. If participants did not remain compliant, their data will not be included in the final analysis. When applicable, participants will be asked for their reasons for poor compliance or dropout. We will collect, assess, and report any spontaneously described adverse events from participants using RedCap data forms. Any unintended or unanticipated adverse events will be reviewed by study physicians for potential medical management. All symptoms will be prospectively followed by phone or in-person until resolution.

### Primary outcome measures

Pain severity, QOL, and physical functioning are key components in a core outcome set for reporting of clinical studies for LBP [36, 37]. Outcome measures will be collected at our biomechanics laboratory during the subjects two baseline visits (week 0) as well as during the two post-intervention visits (weeks 12–13). Table 2 provides the summary of the study outcome measures.

**Table 2:** Summary of outcome measures and participant characteristics. Collected weeks 0–1 (baseline) and 12–13 (post-intervention/wait-list controls)

Primary outcome measures	Data collection instrument or method
Back pain severity	11-point Numerical Pain Rating Scale (NRS_pain_)
	Resting and during activity
Pain impact on quality of life (QOL)	Pain medications used
	Medical Outcomes Short Form-36 (SF-36)
	Oswestry Disability Index (ODI)
	Roland Morris Disability Questionnaire (RMDQ)
Gait kinematics and kinetics	3D motion analysis for temporal spatial parameters of gait and trunk away, bilateral (at self-selected velocity, standard velocity)
	Ground reaction forces
Muscle strength	1-repetition maximum (1-RM) on MedX® clinical Machines: abdominal curl, hip adduction, hip abduction, leg press, and lumbar extension
Muscle morphology	Bilateral B-mode ultrasound measures of muscle thickness and cross-sectional area: multifidus, paraspinals, transversus abdominis, and internal oblique
Step count	StepWatch® monitor, 7-day average count
Participant characteristics	Demographics, weight, Body Mass Index, current medical issues, current medications
	Back pain history
	Pain catastrophizing level (Pain Catastrophizing Scale (PCS))
	Kinesiophobia (11-item Tampa Scale of Kinesiophobia (TSK-11))

### Pain severity and pain impact on QOL

The primary outcome will be the change in the 11-point NRS_pain_ score during rest and activity from baseline to 3 months. The scale has anchors of 0 (no pain) to 10 (worst imaginable pain). The NRS_pain_ is an accepted outcome measure as described in the Initiative on Methods, Measurement, and Pain Assessment in Clinical Trials (IMMPACT) [38]. Participants will rate their current, worst, and best NRS_pain_ scores over the last 24 h, and the scores will be averaged to create the resting NRS_pain_ value [39]. The participants will also be asked to rate their pain severity during walking [40]. The number, type, scheduled nature of the medication and the medicine dose used to self-manage LBP symptoms will be self-reported by participants at baseline and weeks 4, 8, and 12.

One general QOL instrument and two disease-specific instruments will be used to determine the impact of LBP on participant perceptions of QOL [37]. General QOL will be measured by the Medical Outcomes Short Form-36 (SF-36), consisting of eight domain scores and two component scores (Mental, Physical). The SF-36 instrument has been used to track QOL after traumatic injury such as amputation [41]. Higher scores reflect higher QOL. Low back pain-specific QOL will be measured using the modified version of the Oswestry Disability Index (ODI) [42] and the Roland Morris Disability Questionnaire (RMDQ) [43]. The ODI contains 10 questions about the patient-perceived impact of pain on personal care, walking, lifting, sitting, standing, sleeping, sex life, social life, and traveling. The RMDQ consists of 24 items relating to difficulties with daily activities, house work, stair climb, walking, speed of movements, standing, appetite, and sleep. Both instruments are valid, reliable, reproducible, and sensitive to treatment interventions [42, 43]. Higher scores for both instruments reflect greater pain impact and disability due to pain.

### Secondary outcome measures

All of the following secondary outcome measures will be collected the UF Human Dynamics Laboratory in the Orthopaedics and Sports Medicine Institute.

### Kinematics and kinetics of level gait

To assess the effect of HBRX on gait motion, all participants will undergo a 3D gait analysis at baseline and week 12. Upper and lower body kinematic and kinetic data will be collected using a seven-camera, optical motion capture system (Motion Analysis, Inc., Santa Rosa, CA, USA). The camera capture rate will be 120 Hz. Ground reaction forces (GRF) will be collected at 1200 Hz using a force-plated treadmill (AMTI Inc., Watertown, MA, USA) synchronized to the motion capture system. Thirty-three retro-reflective markers will be used to track movement of the body segments from the shoulder to the foot using the marker method of Kadaba et al. [44]. Kinematic data will be processed using Visual 3D software (C-Motion Inc., Rockville, MD, USA). Kinematic variables (joint segment angular velocity, linear velocity, and joint excursion) will be collected from the frontal, sagittal, and transverse planes for ankle, knee, hip, pelvis, shoulder, and elbow. Gait velocity, cadence, step length, vertical displacement of the center of mass, step width, single and double support times, and lateral trunk sway will be calculated. Lateral trunk sway will be calculated using trunk sway in relation to the pelvis [45]. Peak GRF during gait will be calculated and normalized to body weight [46, 47]. Given that GRF and kinematics are velocity-dependent, walking parameters will be collected at two walking velocities, self-selected comfortable velocity and a standard velocity (1.5 m/s) [48]. Data will be expressed as a percentage of the gait cycle.

### Muscle strength

Lower body muscle strength is expected to change with resistance exercise, and changes in strength can potentially affect outcomes of function and pain [49]. Assessments of strength (one-repetition maximum, 1-RM) will be performed as previously described [54] for abdominal curl, hip adductors, hip abductors, leg press, and lumbar extension using MedX® clinical resistance exercise machines. After a brief warm-up in each machine (completing the motion with minimal resistance), progressively higher loads will be provided until the participant can lift the weight stack only one time with good form. Muscle strength will be defined as the 1-RM value for the exercise. Strength measures will be collected at baseline and week 12.

### Muscle morphology

Morphological changes in lumbar and core skeletal muscle will be assessed using a B-mode ultrasound apparatus (Biosound Esaote MyLab 25) with a linear transducer [50]. Changes in lumbar muscle thickness reflect muscle morphology change due to resistance exercise. The ultrasound technique of Blazevich et al. [51] will capture muscle thickness changes in the paraspinal and multifidus muscles. Once the probe has captured thickness, the probe will be rotated by 90° to capture the cross-sectional view. We can consistently obtain paraspinal and multifidus muscle cross-sectional area values (*r* coefficients in our laboratory = 0.088–0.90 for both muscles). Muscle thickness will be measured as described by Wallwork et al. [52]. The participant will be positioned prone on the clinical table with a pillow under the pelvis to minimize lumbar lordosis for both cross-sectional area and muscle thickness measures. The multifidus muscle thickness will be imaged in the longitudinal view allowing for the visualization of the zygapophyseal joints, multifidus muscle bulk, and thoracolumbar fascia [52]. Thickness will be measured at the levels of L2–3 and L4–5 zygapophyseal joints using on-screen calipers later by a blinded tester. Ultrasound measures of muscle thickness and cross-sectional area from lumbar muscle have very strong intraclass correlations with measures obtained using magnetic resonance imaging (MRI; *r* range 0.919–0.970), and can be a surrogate for MRI [50].

Transversus abdominis and internal oblique muscle thickness changes will be measured from the technique described in Belavý et al. [53]. The participants will lie on their back. The ultrasound probe placement for transversus abdominis and internal oblique will be at the closest point between the iliac crest and the inferior angle of the rib cage. The ultrasound probe will be oriented to initially be perpendicular but then rotated along its axis to visualize the transversus abdominis and internal oblique. One image will capture these muscles. The ultrasound tests will be performed at baseline and week 12.

### Daily step count

Step counts have been previously used to objectively estimate community walking activity in persons with chronic LBP [54]. We will collect the number of steps taken over a 7-day period using an ankle-worn StepWatch® step activity monitor (SAM; Cyma, Seattle, WA, USA). StepWatches® will be worn for a week during baseline testing and post intervention. Steps will be collected and received by the study team at the end of every day over the span of the week. More steps per day is a useful facet of participation in activities that can improve overall QOL [55, 56]. The change in step count from baseline to week 12 will be a covariate used in regression analysis for aim 3.

### Participant characteristics

Age, weight, Body Mass Index, current medical issues, current medications, and back pain history will be collected using electronic case report forms. Kinesiophobia and pain catastrophizing can contribute to physical disability in people with chronic pain [20, 57]. Inclusion of kinesiophobia and pain catastrophizing levels will be necessary for statistical analysis in building the model of best fit in for the third study aim. Both kinesiophobia and pain catastrophizing interfere with engagement in exercise and can decrease QOL in persons suffering from chronic pain. Kinesiophobia and pain catastrophizing levels will be measured at baseline using the modified 11-item Tampa Scale of Kinesiophobia (TSK) [58] and the 13-item Pain Catastrophizing Scale (PCS) [59] as covariates. Other variables that change during the study will also be covariates in the statistical analyses.

### Statistical analysis

Statistical analysis will be performed using the Statistical package for the Social Sciences (SPSS v. 24, IBM Corporation, Armonk, NY, USA). An *α* level will be established at 0.05 a priori for all statistical tests. Data will first be examined for normal distribution using the Kolmogorov-Smirnov test, and skewness and kurtosis parameters. Descriptive statistics will be performed to characterize the participants. Means, standard deviation, and frequencies will be obtained for both study groups. The chi-square test for frequency distributions will be used for demographics, and amputation type. Continuous data that are not normally distributed will be transformed prior to analysis.

The main analysis type that will be used to test the hypotheses of aims 1 and 2 will be a mixed-model, repeated measures approach. These analyses will examine the main effects of the exercise treatment on the study outcomes. The independent variables will include study group (HBRX or CON) and time point (baseline, week 12). Dependent variables will include LBP severity, medication use, perceived impact of LBP on QOL (NRS_pain_, medication number, SF-36, ODI, RMDQ) and biomechanical measures (walking velocity, temporal spatial parameters, GRF, key joint angles). Mixed models are the preferred approach to analyze data with repeated measures; these models account for correlation among repeated measurements, flexible time effects, and handle missing data. To test the hypothesis of aim 3, regression analyses will be used to identify significant contributors to the change in pain over 12 weeks. A hierarchical approach will be used to find the model of best fit. Independent variables that will be entered into the models will include changes in lumbar muscle strength, ultrasound measure of lumbar extensor cross-sectional area, and change scores in SF-36, ODI, and RMDQ. In addition to age, sex, and K-classification, models will be adjusted for other participant characteristics if differences are found from baseline to week 12 (Tampa Scale of Kinesiophobia and Pain Catastrophizing Scale scores, daily step number). Subjects who dropped out or missing participant data will not be included in the final analysis, and intention-to-treat will not be utilized. Due to the relatively small study size, actual treatment effects that would be seen may be washed out during an intention-to-treat analysis.

The study team, upon completion of the study, will make the results available in publication format, presented at relevant national scientific conferences and a summary of the results will be provided to each participant, electronically or by mail.

## Discussion

This study will examine the efficacy of a 12-week HBRX program on LBP severity and pain impact in persons with traumatic unilateral transtibial amputation. Measurements of key physiological and mechanical factors before and after exercise training relative to changes in LBP severity will help improve our understanding of pain responsiveness to exercise. To date, exercise training trials in amputees to treat secondary musculoskeletal system conditions, such as low back pain, are scarce. Physical activity-related intervention studies in amputees primarily focus on the earlier stages of post-amputation care for gait retraining [60] and gait improvement [61, 62]. The use of resistance exercise to help correct secondary musculoskeletal pain conditions is novel, and fills an unmet care need of transtibial amputees for long-term health and well-being.

The HBRX intervention adheres to the exercise prescription principles of the ACSM [30, 63]. A unique aspect of this intervention is that participants will be able to perform the exercise in their home environment, free of travel or financial restrictions that are common barriers to long-term success with exercise engagement. The use of Skype®, Facetime, and other platforms to monitor training will provide the first evidence of the feasibility of such a program in the greater amputee community. If the outcomes occur as hypothesized, this study could generate a working, functional model of resistance exercise for chronic LBP in lower limb amputees that could be quickly implemented in various clinical environments.

Determining the contribution of exercise-induced changes in mechanical factors, such as gait asymmetries and loading to LBP responsiveness, will be important for optimizing treatments for amputees. Over half of lower limb amputees suffer from chronic LBP [9], but there are not yet protocols in place to help these patients self-manage this pain. Our statistical procedures will help identify the greatest contributors to changes in NRS_pain_ scores from baseline to week 12. Our findings will guide subsequent interventions to maximize the factors that can have the greatest impact on pain severity for this population. There is a chance that anticipated benefits will not be detected in all study outcomes at the conclusion of the study. There is the possibility that pain does not decrease, but function, gait, and QOL improve. An interpretation could be that gait and compensatory motions after amputation are not primary causes of LBP. The improvement of gait symmetry and mechanical loading are still significant benefits for overall functional QOL. Reduction of movement asymmetries can potentially decrease the risk of other secondary musculoskeletal conditions such as osteoarthritis of the lower extremity joints. There is also the possibility that pain decreases over time, but gait symmetry does not. These findings would indicate that: (1) atrophy-related muscle weakness following amputation facilitates the onset of back pain, and strengthening these muscles is an important therapeutic goal and (2) asymmetrical movement may not be the primary cause of LBP development in amputees. According to prior work, the expected decrease in back pain from resistance exercise will be beneficial for subsequent reduction of back pain-related disability [20], reduction of pain catastrophizing [20], improvement in QOL [21] and increase in steps per day [54]. An opportunity then arises for further research to identify what different types and intensity of exercises may improve gait in lower extremity amputees.

Strengths of the study include a single-assessor-blinded, randomized controlled design. The main study outcomes will be assessed at baseline and at week 12. The outcomes include a combination of objective and subjective measures to capture the exercise adaptations and the patient experience. Objective measures include biomechanical gait parameters, muscle morphology, muscle strength, and daily step count. Subjective, patient-reported outcomes will include pain and its impact on overall QOL. The instruments and tests that will be administered are well-accepted, valid, reliable and sensitive to change [38, 42, 43]. Exercise benefits will be offered to all enrolled participants; the CON group will wait the 12 weeks before beginning the training.

In summary, the findings from this study will help to determine whether a HBRX program for unilateral transtibial amputees with chronic LBP can decrease pain severity and positively impact several physiological and mechanical factors that contribute to LBP. After adjusting for participant characteristics, our analyses will determine the relative contribution of these exercise-induced changes in these factors on pain responsiveness in this population. Future trials can be designed to target the factors we identify here as effective in reducing LBP.

### Trial status

The state of the trial is currently ongoing, and we have not completed patient recruitment at time of submission.

## Additional file

Additional file 1: SPIRIT 2013 Checklist: recommended items to address in a clinical trial protocol and related documents. (DOC 121 kb)

## Abbreviations

1-RM: One-repetition maximum
ACSM: American College of Sports Medicine
CON: Waitlisted Control Group
GRF: Ground reaction forces
HBRX: Home-based resistance exercise group
IMMPACT: Initiative on Methods, Measurement, and Pain Assessment in Clinical Trials
LBP: Low back pain
NRS_Pain_: Numerical Pain Rating Scale
ODI: Oswestry Disability Index
PCS: Pain Catastrophizing Scale
QOL: Quality of life
RISE: Resistance Intensity Scale for Exercise
RMDQ: Roland Morris Disability Questionnaire
TSK: Tampa Scale of Kinesiophobia
UF IRB: University of Florida Institutional Review Board
